# Periodontal Disease Bacteria Specific to Tonsil in IgA Nephropathy Patients Predicts the Remission by the Treatment

**DOI:** 10.1371/journal.pone.0081636

**Published:** 2014-01-28

**Authors:** Yasuyuki Nagasawa, Kenichiro Iio, Shinji Fukuda, Yasuhiro Date, Hirotsugu Iwatani, Ryohei Yamamoto, Arata Horii, Hidenori Inohara, Enyu Imai, Takeshi Nakanishi, Hiroshi Ohno, Hiromi Rakugi, Yoshitaka Isaka

**Affiliations:** 1 Department of Geriatric Medicine and Nephrology, Osaka University, Graduate School of Medicine, Yamada-oka, Suita, Osaka, Japan; 2 Division of Kidney and Dialysis, Department of Internal Medicine, Hyogo College of Medicine, Mukogawa-Cho, Nishinomiya, Japan; 3 Laboratory for Epithelial Immunobiology, RIKEN Research Center for Allergy and Immunology, Suehiro-cho, Tsurumi-ku, Yokohama, Kanagawa, Japan; 4 Graduate School of Nanobioscience, Yokohama City University, Suehiro-cho, Tsurumi-ku, Yokohama, Kanagawa, Japan; 5 Department of Life Science and Medical Bioscience, Waseda University, Wakamatsu-cho, Shinjuku-ku, Tokyo, Japan; 6 Department of Otolaryngology, Osaka University, Graduate School of Medicine, Yamada-oka, Suita, Osaka, Japan; 7 Institute for Advanced Biosciences, Keio University, Mizukami, Kakuganji, Tsuruoka, Yamagata, Japan; University Hospital of the Albert-Ludwigs-University Freiburg, Germany

## Abstract

**Background:**

Immunoglobulin (Ig)A nephropathy (IgAN) is the most common form of primary glomerulonephritis in the world. Some bacteria were reported to be the candidate of the antigen or the pathogenesis of IgAN, but systematic analysis of bacterial flora in tonsil with IgAN has not been reported. Moreover, these bacteria specific to IgAN might be candidate for the indicator which can predict the remission of IgAN treated by the combination of tonsillectomy and steroid pulse.

**Methods and Findings:**

We made a comprehensive analysis of tonsil flora in 68 IgAN patients and 28 control patients using Denaturing gradient gel electrophoresis methods. We also analyzed the relationship between several bacteria specific to the IgAN and the prognosis of the IgAN. *Treponema sp.* were identified in 24% IgAN patients, while in 7% control patients (P = 0.062). *Haemophilus segnis* were detected in 53% IgAN patients, while in 25% control patients (P = 0.012). *Campylobacter rectus* were identified in 49% IgAN patients, while in 14% control patients (P = 0.002). Multiple Cox proportional-hazards model revealed that *Treponema sp.* or *Campylobactor rectus* are significant for the remission of proteinuria (Hazard ratio 2.35, p = 0.019). There was significant difference in remission rates between IgAN patients with *Treponema sp.* and those without the bacterium (p = 0.046), and in remission rates between IgAN patients with *Campylobacter rectus* and those without the bacterium (p = 0.037) by Kaplan-Meier analysis. Those bacteria are well known to be related with the periodontal disease. Periodontal bacteria has known to cause immune reaction and many diseases, and also might cause IgA nephropathy.

**Conclusion:**

This insight into IgAN might be useful for diagnosis of the IgAN patients and the decision of treatment of IgAN.

## Introduction

Immunoglobulin (Ig)A nephropathy (IgAN) is the most common form of primary glomerulonephritis in the world [1]. It is reported to occupy more than a half of the primary glomerulonephritis [2]. It is characterized by IgA deposition to glomerular mesangial cells in pathological point of view and sometimes macroscopic hematuria after upper respiratory infection in clinical point of view. Since it was firstly reported by Berger in 1968 [3], the etiology and cause of the disease has been an ultimate mystery [4], although several genes [5], [6] or single nucleotide polymorphism [7]–[12] were reported to be associated with incidence or progression of IgA nephropathy. In a couple of decades ago, its prognosis was believed to be rather good, but Koyama et al reported that 39% of the patients with IgAN end up in dialysis or death in 20 years [13], [14]. Recently our group reported that 61% of IgAN patients with smoking reached to the 150% elevation of creatine in 15 years [15], [16]. Moreover, the occurrence is often seen in the younger generations such as in their teens or twenties, the treatment strategy focusing on the longer term is being a crucial problem.

It is also well known that in upper respiratory infection such as tonsillitis, IgAN patients often manifest the deterioration of urinary findings; macroscopic hematuria [17]. Therefore, tonsillectomy is a focus of much attention in treating IgAN. In the wake of this trend, combination of tonsillectomy and steroid pulse therapy was reported to be effective [18]–[20] and tonsillectomy alone was reported to have a favorable effect to the IgAN [21]. The indicators which can predict the remission of IgAN by the combination therapy become desired in the case of application of this treatment [22], because there were not any confirmative clinical marker for decision of treatment [23]–[25]. The gene expression and structural difference between the tonsils in patients with IgAN and those in control were reported [26], [27]. The key factor which induced these kinds of differences in tonsils of IgAN patients may be infection. Some bacteria were reported to be the candidate of the antigen or the pathogenesis of IgAN [28]–[30], but systematic analysis of bacterial flora in tonsil with IgAN has not been reported. Moreover, these bacteria specific to IgAN might be candidate for the indicator which can predict the remission of IgAN.

We performed tonsillectomy combined with steroid pulse as a treatment to IgAN,and we made a comprehensive analysis of tonsil flora in IgAN patients using Denaturing gradient gel electrophoresis (DGGE) methods. We also analyzed the relationship between several bacteria specific to the IgAN and the prognosis of the IgAN.

## Materials and Methods

### Patients Enrollment

Tonsil tissues of the IgAN patients were obtained from 104 consecutive patients undergoing tonsillectomy at Osaka university hospital from April, 2004 to January, 2008. A pair of palatine tonsils was resected when tonsillectomy. One of tonsils was used for clinical histological evaluation. Another of tonsils was used for this study. More than half of the tonsil was used for RNA extraction. The diagnosis of the IgAN in these patients was made by the renal biopsy. The 28 control tonsil tissues were obtained from the patients suffering from chronic tonsillitis at tonsillectomy. Inclusion criteria for DGGE analysis in IgAN patients comprised age 18 to 65 years, serum creatinine at tonsillectomy of  = <2.0 mg/dl, and urinary protein > = 0.3 g/gCr. Exclusion criteria comprised use of steroid and/or other immunosuppressive agents at tonsillectomy. Accordingly, 68 patients were eligible for DGGE analysis. For survival analysis in IgAN patients, we excluded patients of follow up period <6 months, and patients without hematuria at tonsillectomy. Therefore, 59 patients were eligible for survival analysis. IgAN patients received tonsillectomy and 7 days later received intravenous steroid pulse therapy (methylprednisolone pulses of 500 mg/day) for three days, followed by oral prednisolone at an initial dose of 1 mg/kg for 11days. Then, patients received intravenous steroid therapy again for three days, followed by oral prednisolone at a dose of 30 mg/day and gradually tapered by 5–10 mg/1–2 months and discontinued within about one year. This treatment protocol was slightly weaker than the protocol used by Pocci et al [25], [31], [32]. Remission of urinary protein and that of urinary occult blood were defined as negative or almost negative, twice by dipstick at least in one month interval.

Written informed consent was obtained from all participating subjects. This study was approved by the ethical committee of Osaka University Graduate School of Medicine.

### PCR-DGGE

In order to evaluate the bacterial flora in tonsil, we performed DGGE analysis using cDNA. The tonsil tissue was homogenized; total RNA was extracted using TRIzol (Invitrogen, Carlsbad, CA, US) and 0.4 microgram of RNA was converted to cDNA using random primers and SuperScript II (Invitrogen, Carlsbad, CA). For PCR-DGGE analyses, each cDNA sample was amplified by PCR with universal bacterial primers 954f (cgcccgccgcgccccgcgcccggcccgccgcccccgccccgcacaagcggtggagcatgtgg) and 1396r (GCCCGGGAACGTATTCACCG) specific for V6 to V8 regions of the 16S rRNA gene [33]. The reaction mixtures and PCR conditions were referred to previous report [34].

After confirmation of the PCR product with agarose gel electrophoresis, DGGE was performed with the DCode universal mutation detection System (Bio-Rad laboratories). Polyacrylamide gel conditions for denaturing gradient, migration and differentiation were referred to previous report [33]. The electrophoresis was conducted with constant voltage of 82 V at 60°C for 15 h. Gels were stained with SYBR Green I (Lonza, Rockland, ME), and acquired by GelDoc XR (Bio-Rad laboratories).

### Statistical analysis of DGGE image

The DGGE image was read by Quantity One software (BioRad) and the intensity and position of bands in each lanes ware read into a spectrum of 100 variables. Partial least squares discriminant analysis (PLS-DA), a regression extension of the classical Principal Component Analysis appropriate to our dataset, was run with the R software using the pls package (ver 2.0) and the “simpls” method [35]. Briefly, the DGGE band dataset was imported into the R software, and variance and regression were computed in a class-supervised manner and principal components (PC) scores. Data were visualized as PC score plots, with the PC1 axis exhibiting most of the differences among the samples, while PC2 and PC3 corresponded to factors with decreasing contribution to the differences. Each coordinate on the scores plot represents an individual sample and each coordinate on the loadings plots represents DGGE gel position of bands. Thus, the loadings plots provide information on band position responsible for the position of coordinates or clusters of samples in the corresponding scores plots.

### Identification of bacterial origin

There were 3 bands in the DGGE analysis which characterized the flora in tonsils of the patients with IgAN. For identification of DNA sequences of bacterial origin in the gel, selected DGGE bands were excised from original gels and their DNA fragments were reamplified with corresponding primers. The obtained PCR product was purified, cloned by TA cloning method using TA cloning Kit as manufacture's instruction (INVITOROGEN, Life Technologies, Carlsbad, CA). The clones were sequenced as described in previous report [34]. The sequences were submitted to BLAST search programs in DDBJ (DNA Data Bank of Japan) to determine their closest relatives.

### Pathological evaluation

Histological evaluation was performed according to the Oxford classification [36], [37]. The four pathological variables of Oxford classification were evaluated: mesangial hypercellularity score≤0.5 or >0.5, presence or absence of segmental sclerosis or adhesion, presence or absence of endocapillary hypercellularity, and tubular atrophy/interstitial fibrosis ≤25%, 26–50%, or >50%.

### Statistical analysis

Normally distributed continuous variables were expressed as mean±SD, and non-normally distributed continuous variables as median (interquartile range). Categorical variables were expressed as numbers (proportions). For comparison between two groups, the t-test was used for normally distributed continuous variables, the Mann-Whitney test for non-normally distributed continuous variables, and χ^2^ test for categorical variables. Kaplan-Meier analysis using log-rank test was used to compare survival rate. We used the Cox proportional hazards model to assess the impact of covariates for the remission of urinary protein and urinary occult blood. The results of the analyses are expressed as hazards ratios with 95% confidence intervals and a P value. P values less than 0.05 were considered statistically significant. All statistical analyses were performed using JMP for windows version 8.0.1 (SAS Institute Inc., Cawy, NC, US).

## Results

Clinical characteristics of 68 IgA nephropathy patients and 28 control patients are presented in Table 1. Mean glomerular filtration rate (GFR) in IgA nephropathy patients was 85 ml/min, while mean GFR in control group was 124 ml/min. GFR was calculated using GFR estimated equation [38]. Urinary protein in IgA nephropathy patients was 0.59 (0.38 to 1.04) g/day. There were no significant difference except serum creatinine and eGFR between IgA nephropathy patients and control patients.

**Table 1:** Patient characteristics performed by DGGE analysis.

Baseline characteristics	IgAN group (n = 68)		Control group (n = 28)		P
Age (year)	32	(27–46)	29	(22–40)	0.107
Female [n(%)]	36	/68 (53)	13	/28 (46)	0.896
Systolic blood pressure (mmHg)	113	±14	112	±14	0.647
Urinary protein (g/gCr)	0.59	(0.38–1.04)		-	-
Serum creatinine (mg/dl)	0.9	(0.7–1.1)	0.7	(0.6–0.9)	0.002*
eGFR (ml/min/1.73 m^2^)	85	±29	124	±40	<0.001*
Serum IgA (mg/dl)	316	(238–381)	297	(215–392)	0.573
Serum C3 (mg/dl)	127	±24	130	±28	0.688
CRP (mg/dl)	0	(0–0.2)	0	(0–0.2)	0.276

DGGE, Denaturing Gradient Gel Electrophoresis; eGFR, estimated glomerular filtration rate.

Data are expressed as mean ± SD, median (interquartile range).

* P<0.05 by unpaired t test, Wilcoxon signed-ranks test or χ^2^ test.

**Figure 1:**
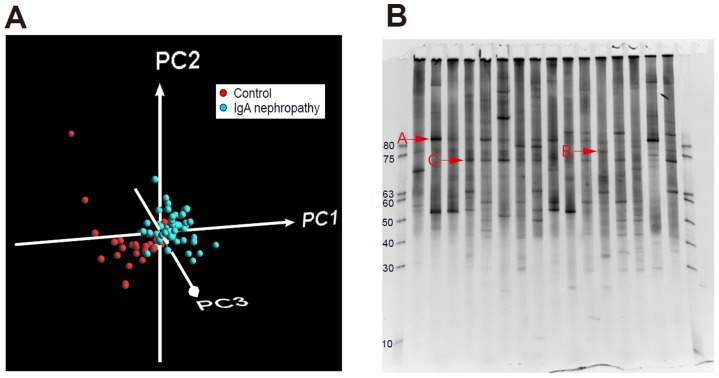
Comprehensive analysis of tonsil flora of IgA nephropathy patients compared with those of control patients by denaturing gradient gel electrophoresis (DGGE) method (A) Partial least squares-discriminant analysis (PLS-DA) on tonsil-associated bacterial composition in control and IgA nephropathy patient: Result of PLS-DA on DGGE band data set of each control (red) and IgA nephropathy patient (blue) are shown (n = 68 and 28, respectively). Proportions of the first (PC1), second (PC2), and third (PC3) components are 50.1%, 13.3%, and 3.78%, respectively. **(B) Results of PCR-Denaturing gradient gel electrophoresis (DGGE) analysis:** Specific DGGE bands in IgA nephropathy patients were shown as A, B, and C.

All samples including 68 tonsils with IgAN patients and 28 tonsils with control patients were analyzed by PCR-DGGE method followed by PLS-DA. The patterns of the bands in DGGE analysis were scanned and PLS-DA was performed based on the intensity and position of the bands in each lane as shown in Figure 1A. Based on the PLS-DA, 3 bands strongly contributed to the feature of the IgAN, which were shown in original gel in Figure 1B.

These 3 bands were cloned and Bacteria A, B, and C shown in Figure 1B were identified as *Treponema* sp., *Haemophilus segnis*, and *Campylobacter rectus*, respectively. *Treponema sp.* were identified in 24% IgAN patients, while the bacteria were identified in 7% control patients (P = 0.062). *Haemophilus segnis* were detected in 53% IgAN patients, while the bacteria were identified in 25% control patients (P = 0.012). *Campylobacter rectus* were identified in 49% IgAN patients, while the bacteria were identified in 14% control patients (P = 0.002).

The IgAN patients were divided by the presence or absence of the each bacterium, and the patient characteristics were compared between two groups in each bacterium (Table 2). Basically there were no significant difference between IgA nephropathy patients with bacterium and those without bacterium.

**Table 2:** Patient characteristics in each bacterial flora.

Baseline characteristics	IgAN all (n = 59)	*Treponema sp.*		*Haemophilus segnis*		*Campylobacter rectus*	
		Positive (n = 12)	Negative (n = 47)	Positive (n = 31)	Negative (n = 28)	Positive (n = 26)	Negative (n = 33)
Age (year)	32 (26–46)	36 (24–43)	31 (26–46)	32 (23–45)	32 (27–46)	31 (27–48)	33 (23–44)
Female [n(%)]	33/59(56)	6/12(50)	27/47(57)	17/31(55)	16/28(57)	14/26(54)	19/33(58)
Systolic blood pressure (mmHg)	113±14	119±12	111±14	110±15	115±13	113±13	113±15
Use of RAS blockade [n(%)]	29/59(49)	5/12(42)	24/47(51)	15/31(48)	14/28(50)	12/26(46)	17/33(52)
Urinary protein (g/gCr)	0.60 (0.40–0.99)	0.46 (0.30–0.46)	0.66 (0.43–1.08)	0.66 (0.42–0.94)	0.53 (0.38–1.06)	0.51 (0.40–0.88)	0.66 (0.40–1.04)
Serum creatinine (mg/dl)	0.9 (0.7–1.1)	0.8 (0.7–0.9)	0.9 (0.7–1.1)	0.9 (0.7–1.1)	0.9 (0.7–1.1)	0.8 (0.7–1.1)	0.9 (0.7–1.1)
eGFR (ml/min/1.73 m^2^)	87±29	93±27	86±30	89±30	85±29	88±30	86±30
Serum total cholesterol (mg/dl)	196 (182–235)	216 (188–256)	195 (176–232)	191 (178–226)	210 (183–246)	195 (184–223)	198 (173–246)
Serum IgA (mg/dl)	300 (230–367)	347 (220–404)	299 (231–349)	300 (239–372)	296 (220–349)	320 (242–371)	288 (219–348)
Serum C3 (mg/dl)	126±24	128±19	125±25	127±25	125±23	119±20	131±25

RAS, renin angiotension system; eGFR, estimated glomerular filtration rate;

Data are expressed as mean ± SD, median (interquartile range).

Univariate Cox proportional-hazards model for proteinuria revealed that the *Campylobacter rectus* is the significant factor for the remission of proteinuria (Hazard ratio 1.96, p = 0.041) along with the urinary protein level as known before. *Treponema sp.* is the marginally significant factor for the remission of proteinuria (Hazard ratio 2.08, p = 0.067). *Campylobacter rectus* or *Treponema sp.* is the strong factor for the remission of proteinuria (Hazard ratio 2.35, p = 0.011). Multiple Cox proportional-hazards model for proteinuria also revealed that *Treponema sp.* is significant factor for the remission of proteinuria along with proteinuria in Model 1 in table 3 (hazard ratio 2.62, p = 0.034), and that *Campylobacter rectus* is marginally significant factor (Hazard ratio 1.86, p = 0.066). *Treponema sp.* or *Campylobactor rectus* are significant for the remission of proteinuria along with urinary protein in Model 2 in Table 3 (Hazard ratio 2.35, p = 0.019).

**Table 3:** Multivaliate Cox proportional-hazards regression model for urinary protein remission rate.

	Model 1			Model 2		
Baseline characteristics	HR	95% CI	P-value	HR	95% CI	P-value
Age (per 10 year of age)	1.04	(0.75 to 1.44)	0.825	1.08	(0.79 to 1.47)	0.644
Female (versus male)	0.65	(0.31 to 1.33)	0.236	0.71	(0.35 to 1.46)	0.354
Systolic blood pressure (per 10 mmHg)	0.87	(0.65 to 1.16)	0.334	0.90	(0.69 to 1.18)	0.434
Use of RAS blockade	0.94	(0.45 to 1.96)	0.870	0.91	(0.44 to 1.88)	0.796
Urinary protein (per 1.0 g/gCr)	0.55	(0.31 to 0.97)	0.039*	0.56	(0.32 to 1.00)	0.051
eGFR (per 10 ml/min/1.73 m^2^)	0.95	(0.82 to 1.10)	0.531	0.96	(0.84 to 1.11)	0.625
Bacterial flora detected by DGGE analysis						
*Treponema sp.*	2.62	(1.07 to 6.38)	0.034*	-	-	-
*Haemophilus segnis*	1.39	(0.67 to 2.87)	0.377	1.18	(0.61 to 2.30)	0.625
*Campylobacter rectus*	1.86	(0.96 to 3.60)	0.066	-	-	-
*Treponema sp.* or *Campylobacter rectus*	-	-	-	2.35	(1.15 to 4.78)	0.019*

HR, hazard ratio; CI, confidence interval; RAS, renin angiotension system; eGFR, estimated glomerular filtration rate; DGGE, Denaturing Gradient Gel Electrophoresis.

* Statistically significant.

**Figure 2:**
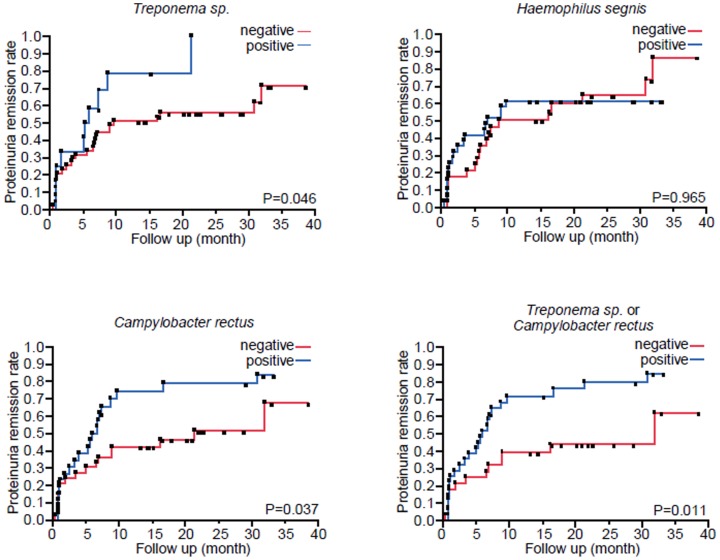
Associations between urinary protein remission and distinct kinds of bacteria; *Treponema sp.*, *Haemophilus segnis*, *Campylobacter rectus*, and *Treponema sp.* or *Campylobacter rectus*. Differences in urinary protein remission with or without bacterial flora were compared using Kaplan-Meier curves and tested using log-rank. P<0.05 was considered to be statistically significant.

The remission rates of proteinuria between patients with bacteria and those without bacteria were analyzed by Kaplan-Meier analysis using log-rank test (Figure 2).

**Figure 3:**
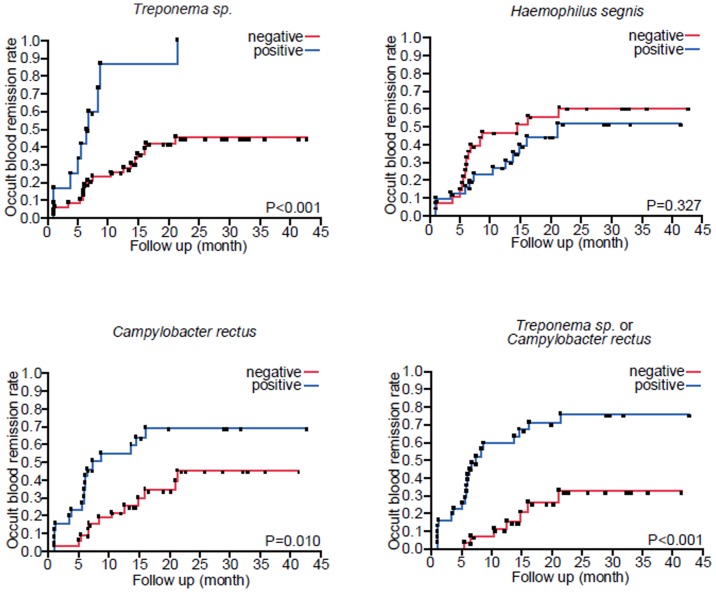
Associations between urinary occult blood remission and distinct kinds of bacteria; *Treponema sp.*, *Haemophilus segnis*, *Campylobacter rectus*, and *Treponema sp.* or *Campylobacter rectus*. Differences in urinary occult blood remission with or without bacterial flora were compared using Kaplan-Meier curves and tested using log-rank. P<0.05 was considered to be statistically significant.

Univariate Cox proportional-hazards model for hematuria revealed that the *Campylobacter rectus* is the significant factor for the remission of occult blood (Hazard ratio 2.35, p = 0.029). *Treponema sp.* is also the significant factor for the remission of hematuria (Hazard ratio 4.27, p = 0.004). *Campylobacter rectus* or *Treponema sp.* are the strong factor for the remission of hematuria (Hazard ratio 4.54, p<0.001). Multiple Cox proportional-hazards model for hematuria also revealed the similar results as the model for proteinuria (Table 4). The remission rates of hematuria were also analyzed by Kaplan-Meier analysis using log-rank test. The results were similar to the results of the remission rate of proteinuria (Figure 3).

**Table 4:** Multivaliate Cox proportional-hazards regression model for urinary occult blood remission rate.

	Model 1			Model 2		
Baseline characteristics	HR	95% CI	P-value	HR	95% CI	P-value
Age (per 10 year of age)	0.90	(0.61 to 1.28)	0.569	0.96	(0.68 to 1.34)	0.798
Female (versus male)	1.10	(0.53 to 2.71)	0.820	1.45	(0.65 to 3.26)	0.366
Systolic blood pressure (per 10 mmHg)	0.95	(0.96 to 1.03)	0.766	0.99	(0.74 to 1.33)	0.953
Use of RAS blockade	0.98	(0.42 to 2.46)	0.970	0.84	(0.37 to 1.93)	0.685
Urinary protein (per 1.0 g/gCr)	0.89	(0.55 to 1.34)	0.614	0.97	(0.61 to 1.54)	0.882
eGFR (per 10 ml/min/1.73 m^2^)	0.97	(0.82 to 1.15)	0.718	0.98	(0.83 to 1.16)	0.822
Bacterial flora detected by DGGE analysis						
*Treponema sp.*	4.27	(1.43 to 10.55)	0.004*	-	-	-
*Haemophilus segnis*	1.07	(0.45 to 2.52)	0.878	0.91	(0.42 to 1.95)	0.800
*Campylobacter rectus*	2.35	(0.98 to 4.68)	0.029*	-	-	-
*Treponema sp.* or *Campylobacter rectus*	-	-	-	4.54	(1.85 to 11.12)	0.001*

HR, hazard ratio; CI, confidence interval; RAS, renin angiotension system; eGFR, estimated glomerular filtration rate; DGGE, Denaturing Gradient Gel Electrophoresis.

* Statistically significant.

To know whether these bacteria might be related to pathological features which were important predictor of renal prognosis, we evaluated the association between bacteria detected by DGGE analysis and histopathological factors. We scored 30 patients who were performed renal biopsy within a year of tonsillectomy by using oxford classification and compared the scores with clinical features and bacteria. The prevalence of each component of Oxford classification was summarized in Table S1 in Supplementary Tables. We found that there was no relationship between bacteria and histological factors (Table S2, S3 in Supplementary Tables).

## Discussion

In this study, we comprehensively compared the bacterial flora of the tonsil between IgAN and chronic tonsillitis patients using PCR-DGGE and PLS-DA, and revealed that Treponema sp., Haemophilus segnis, and Campylobacter rectus are specific to IgAN patients. Moreover, we found the remission rates of proteinuria and hematuria were significantly related with the prevalence infection of Treponema sp. and Campylobacter rectus, both of which were reported to be the cause of periodontal disease [39]–[41], while the existence of Haemophilus segnis had no relationship with clinical course. This result suggests that the detection of bacteria by DGGE analysis could predict the therapeutic effect of tonsillectomy and steroid pulse therapy, and they might reflect the abnormal mucosal immunity in tonsil.

One of clinical manifestations of the IgAN is the macrohematuria soon after the tonsillitis. This feature indicated the relationship between infection in tonsils and IgAN. *Haemophilus parainfluenzae* was reported to be more commonly isolated from the pharynx of patients with IgAN than from those with other diseases [28]. In this studies, *Haemophilus parainfluenzae* was confirmed by cultural and antibody methods. These methods could detect the *Haemophilus segnis as Haemophilus parainfluenzae*, because of antigen similarity [42]. In our study, tonsils with 53% of IgA patients might be infected by *Haemophilus segnis* which are same as the previous reports [28], [43]. There is no report that the relationship between *Haemophilus parainfluenzae* and the prognosis of IgAN, which is compatible with our results.

*Treponema sp.* and *Campylobacter rectus* were newly detected bacteria to be associated with IgAN in this report. These bacteria belong to the anaerobic bacterium species and were reported to be the cause of periodontal disease [39]–[41]. This is the reason why these two bacteria could not be detected by the usual culture method in the previous reports in IgAN research [28]. The method we employed in this study is DGGE method, which make it possible for us to detect comprehensive bacterial flora, including anaerobic bacteria. In the young generation, especially in babyhood, human beings are usually free from periodontal bacteria, and also free from IgAN. *Campylobacter rectus* get dominant from the age of nine in the periodontal area, and *Treponema denticola* and *Treponema forsythensis* gets dominant from the age of five [44]. Kappa statistic analysis between these periodontal bacteria in mother and those in children showed high value, suggesting that mother and children often have the same periodontal bacteria [44]. This might explain that IgAN sometimes can be seen in the same family, although the gene related with IgAN can also partially explain this phenomenon [45].

These are two interpretations of the relationship between those three bacteria and IgAN. One possibility is that these three bacteria have causality of IgAN. The membrane antigens of *Haemophilus parainfluenzae* was reported to induce the IgAN in mice from 30 to 40 weeks of age [46]. Our data suggested that *Treponema sp.* and *Campylobacter rectus* which could cause periodontal disease have stronger association with IgAN, so there is possibility that these two bacteria might have stronger causality of IgAN than *Haemophilus parainfluenzae*. Periodontal disease was reported to have an interaction between bacterial infection in periodontal area and the degree of the systemic inflammatory response [47]. In addition, the activation of the toll like receptor (TLR) 9 which recognizes bacterial CpG-DNA was reported to affect the severity of IgAN [48], and constitutive TLR signaling by intestinal commensal microflora caused glomerulonephritis [49]. These reports speculate that interaction between host immune system and bacteria, which cause constitutive stimulation of TLR in the tonsil, might cause IgAN.

The second possibility is that these bacteria are simply associated with IgAN. Tonsil in the patients with IgAN had several features, such as structural changes [27] and gene expression change [26], resulting in the change of immune response [26], [50]–[52]. Those immune changes in tonsil might allow the specific bacteria to grow in tonsil, such as these three bacteria. In this explanation, the existence of these thee bacteria might be a result of the tonsil condition in IgAN.

Recently many reports suggested that periodontal disease associates with atherosclerosis diseases such as coronary artery disease [53]–[58] and progression of chronic renal disease [59], [60]. Basically, the relationship between atherosclerosis diseases and periodontal diseases are explained by the inflammatory mechanism [56]. There are also several reports suggesting the relationship between the periodontal disease and autoimmune disease such as rheumatoid arthritis [61], [62]. This relationship was explained by autoimmune response to the periodontal bacteria [63]. The periodontal bacteria were directly detected in athermanous plaque [64], [65]. The component of streptococcus was reported to be directly involved in hemorrhage stroke [64]. These reports suggested the periodontal disease might have more pathogenicity than chronic inflammatory response. Moreover, some periodontal pathogen directly altered T-cell response [66]. Our data suggested that the periodontal bacteria might have strong association with IgAN, one of most common renal disease rather than with histological components. The continued infections by these periodontal bacteria might stimulate IgA production by T cells in tonsil, resulting in the IgA which has some errors in their glycosylation. The IgA with abnormal glycosylation had been reported to have ability to bind to the glomeruli in IgA nephropathy [4], [67]. The tonsillectomy and steroid pulse might normalize the IgA production and glycosylation of IgA [68], resulting in the remission of IgA nephropathy. Therefore, these bacteria might be associated with IgA nephropathy and its clinical course after treatment.

In this study, there are several limitations. First, this study is designed as the retrospective manner. Prospective study should be designed to confirm these results, although the tonsils from IgA patients were obtained before the clinical outcomes including urinary protein and hematuria are confirmed. Second, it is hard to distinguish causality from association according to the relationship between the bacteria specific to IgAN and clinical course, as described above. Further study is required to confirm that these bacteria might cause IgAN in experimental mice. However, this study showed that these bacteria are located at least close to the origin of IgAN. Third, *Treponema sp.* includes many species. According to our data, *Treponema sp.* might be *Treponema denticola*, but it is not confirmative. Although it is hard to identify the bacteria from *Treponema sp.*, the basic relationship between the bacteria and the clinical course or IgAN itself is confirmative from our data. Forth, there is some possibility that some unknown factors might attenuate the PCR efficacy in DGGE analysis of tonsil flora, although the primers which used in our study are standard primer in this method [33]. There was no report which confirmed the PCR efficacy in DGGE method in tonsil flora analysis, although we compared the results of DGGE analysis in tonsils with IgA nephropathy to those with tonsillitis, so the effect of unknown factors should be counterbalanced by the comparison step.

In conclusion, *Treponema sp.*, *Haemophilus parainfluenzae*, *Campylobacter rectus* are specific to the tonsils in patients with IgAN. The existence of *Treponema sp* and *Campylobacter rectus* in tonsil is the strong and significant indicator of remission of IgAN treated with tonsillectomy and steroid pulse. This insight into IgAN might be useful for diagnosis of the IgAN patients and the decision of treatment of IgAN.

## Supporting Information

File S1 Table S1. Pathologic features in patients diagnosed IgA nephropathy by renal biopsy within a year of tonsillectomy (n = 30). Table S2. Clinical characteristics in patients diagnosed IgA nephropathy by renal biopsy within a year of tonsillectomy according to mesangial hypertrophy and endocapillary hypercellularity. Table S3. Clinical characteristics in patients diagnosed IgA nephropathy by renal biopsy within a year of tonsillectomy according to segmental glomerulosclerosis and tubular atrophy/interstitial fibrosis.
(DOCX)
