# Supplementary material for: Periodontal Disease Bacteria Specific to Tonsil in IgA Nephropathy Patients Predicts the Remission by the Treatment
Source: PLoS One. 2014 Jan 28;9(1):e81636. doi: 10.1371/journal.pone.0081636 (PMC3904818; doi:10.1371/journal.pone.0081636)
Supplement: File S1 — Table S1. Pathologic features in patients diagnosed IgA nephropathy by renal biopsy within a year of tonsillectomy (n = 30). Table S2. Clinical characteristics in patients diagnosed IgA nephropathy by renal biopsy within a year of tonsillectomy according to mesangial hypertrophy and endocapillary hypercellularity. Table S3. Clinical characteristics in patients diagnosed IgA nephropathy by renal biopsy within a year of tonsillectomy according to segmental glomerulosclerosis and tubular atrophy/interstitial fibrosis. (DOCX) [file pone.0081636.s001.docx]

**Table S1. Pathologic features in patients diagnosed IgA nephropathy by renal biopsy within a year of tonsillectomy (n=30)**

|  | n (%) |
| --- | --- |
| Mesangial hypercellularity score |  |
| ≤0.5 | 16(53) |
| >0.5 | 14(47) |
| Endocapillary hypercellularity |  |
| Absent | 21(70) |
| present | 9(30) |
| Segmental glomerulosclerosis |  |
| Absent | 7(23) |
| Present | 23(77) |
| Tubular atrophy and intestinal fibrosis |  |
| 0 to 25% | 28(93) |
| 26 to 50% | 2 (7) |
| 51% + | 0(0) |

**Table S2. Clinical characteristics in patients diagnosed IgA nephropathy by renal biopsy within a year of tonsillectomy according to mesangial hypertrophy and endocapillary hypercellularity**

|  | Mesangial hypercellularity score | | | Endocapillary hypercellularity | | |
| --- | --- | --- | --- | --- | --- | --- |
|  | ≤0.5 | >0.5 | P | absent | present | P |
| Age (year) | 31  (20–40.5) | 27.5  (25-32) | 0.454 | 32  (21-41) | 29  (27-30) | 0.751 |
| Female [n(%)] | 10/16  (63) | 6/14  (43) | 0.282 | 12/21  (57) | 4/9  (44) | 0.523 |
| Systolic blood pressure (mmHg) | 112 ± 13 | 118 ± 15 | 0.335 | 116 ± 16 | 112 ± 9 | 0.558 |
| Use of RAS blockade　[n(%)] | 6/16  (38) | 6/14  (43) | 0.765 | 6/21  (29) | 6/9  (67) | 0.051 |
| Urinary protein (g/gCr) | 0.49  (0.31-1.31) | 0.68  (0.43-1.21) | 0.506 | 0.49  (0.38-0.71) | 0.88  (0.64-1.21) | 0.081 |
| eGFR (ml/min/1.73m^2^) | 96 ± 26 | 89 ± 29 | 0.508 | 94 ± 29 | 90 ± 25 | 0.717 |
| Bacterial flora detected by DGGE analysis |  |  |  |  |  |  |
| *Treponema sp.* | 3/16  (19) | 5/14  (36) | 0.295 | 6/21  (29) | 2/9  (22) | 0.719 |
| *Haemophilus segnis* | 9/16  (56) | 7/14  (50) | 0.732 | 13/21  (62) | 3/9  (33) | 0.151 |
| *Campylobacter rectus* | 7/16  (44) | 8/14  (57) | 0.464 | 11/21  (52) | 4/9  (44) | 0.690 |

RAS, renin angiotension system; eGFR, estimated glomerular filtration rate; DGGE, Denaturing Gradient Gel Electrophoresis;

Data are expressed as mean ± SD, median (interquartile range).

**Table S3. Clinical characteristics in patients diagnosed IgA nephropathy by renal biopsy within a year of tonsillectomy according to segmental glomerulosclerosis and tubular atrophy/ interstitial fibrosis**

|  | Segmental glomerulosclerosis | | | Tubular atrophy/interstitial fibrosis | | | |
| --- | --- | --- | --- | --- | --- | --- | --- |
|  | absent | present | P | 0 to 25% | 26 to 50% | >51% | P |
| Age (year) | 21  (19-33) | 30  (27-39) | 0.056 | 30  (22-38.5) | 28.5  (28-29) | - | 0.771 |
| Female [n(%)] | 4/7  (57) | 12/23  (52) | 0.818 | 15/28  (54) | 1/2  (50) | 0/0 | .922 |
| Systolic blood pressure (mmHg) | 116 ± 18 | 114 ± 13 | 0.802 | 114 ± 14 | 120 ± 14 | - | 0.598 |
| Use of RAS blockade　[n(%)] | 2/7  (29) | 10/23  (43) | 0.481 | 10/28  (36) | 2/2  (100) | 0/0 | 0.073 |
| Urinary protein (g/gCr) | 0.47  (0.23-0.52) | 0.66  (0.39-1.45) | 0.249 | 0.51  (0.39-1.07) | 2.16  (0.71-3.60) | - | 0.135 |
| eGFR (ml/min/1.73m^2^) | 109 ± 22 | 88 ± 27 | 0.065 | 95 ± 26 | 55 ± 5 | - | 0.042* |
| Bacterial flora detected by DGGE analysis |  |  |  |  |  |  |  |
| *Treponema sp.* | 1/7  (14) | 7/23  (30) | 0.398 | 8/28  (29) | 0/2  (0) | - | 0.377 |
| *Haemophilus segnis* | 5/7  (71) | 11/23  (48) | 0.273 | 15/28  (54) | 1/2  (50) | - | 0.922 |
| *Campylobacter rectus* | 4/7  (57) | 11/2 (48) | 0.666 | 14/28  (50) | 1/2  (50) | - | 1.000 |

RAS, renin angiotension system; eGFR, estimated glomerular filtration rate; DGGE, Denaturing Gradient Gel Electrophoresis;

Data are expressed as mean ± SD, median (interquartile range).

*Statistically significant
